# The Neurocircuitry of fluid satiation

**DOI:** 10.14814/phy2.13744

**Published:** 2018-06-21

**Authors:** Philip J. Ryan

**Affiliations:** ^1^ Florey Institute of Neuroscience and Mental Health Parkville Victoria Australia

**Keywords:** Area postrema, Calcium imaging, DREADDs (designer receptors exclusively activated by designer drugs), Fluid satiation, Lamina terminalis, Neurocircuitry, Nucleus of the solitary tract, Optogenetics, Parabrachial nucleus, Postabsorptive inputs, Preabsorptive inputs

## Abstract

Fluid satiation, or quenching of thirst, is a critical homeostatic signal to stop drinking; however, its underlying neurocircuitry is not well characterized. Cutting‐edge genetically encoded tools and techniques are now enabling researchers to pinpoint discrete neuronal populations that control fluid satiation, revealing that hindbrain regions, such as the nucleus of the solitary tract, area postrema, and parabrachial nucleus, primarily inhibit fluid intake. By contrast, forebrain regions such as the lamina terminalis, primarily stimulate thirst and fluid intake. One intriguing aspect of fluid satiation is that thirst is quenched tens of minutes before water reaches the circulation, and the amount of water ingested is accurately calibrated to match physiological needs. This suggests that ‘preabsorptive’ inputs from the oropharyngeal regions, esophagus or upper gastrointestinal tract anticipate the amount of fluid required to restore fluid homeostasis, and provide rapid signals to terminate drinking once this amount has been consumed. It is likely that preabsorptive signals are carried via the vagal nerve to the hindbrain. In this review, we explore our current understanding of the fluid satiation neurocircuitry, its inputs and outputs, and its interconnections within the brain, with a focus on recent studies of the hindbrain, particularly the parabrachial nucleus.

## Introduction

Fluid satiation is a key homeostatic signal which limits the amount of fluid ingested and prevents the potentially lethal consequences of overhydration, such as severe hyponatremia and cerebral edema, which can occur in patients with psychogenic polydipsia (Goldman 2014). Tight control of fluid homeostasis is vital for maintaining cell size which is required for proper cell functioning; and for maintaining stable blood volume and pressure, which are necessary for transporting essential nutrients and oxygen around the body (Leib et al. 2016).

The sensation or ‘homeostatic emotion’ of fluid satiation is more than just the absence of thirst, at least initially. Based on human reports, fluid satiation is inherently pleasant, particularly after extreme dehydration (Saker et al. 2014). By contrast, thirst is generally aversive, and animals will work to suppress the negative valence associated with thirst (Allen et al. 2017). The pleasure associated with fluid satiation gradually diminishes as more water is consumed; and if water is drunk to excess, the sensation to stop drinking becomes aversive (Saker et al. 2014). These observations suggest that bodily signals of pleasure and aversion help control the amount of fluid intake during drinking bouts.

One intriguing aspect of fluid satiation is that thirst is quenched tens of minutes before water reaches the circulation – well before fluid homeostasis has been corrected – and the amount of water ingested is accurately calibrated to match physiological needs (Zimmerman et al. 2016). This suggests that ‘preabsorptive’ inputs from the oropharyngeal regions, esophagus or upper gastrointestinal tract anticipate the amount of fluid required to restore fluid homeostasis, and provide rapid signals to terminate drinking once this amount has been consumed (Gibbs et al. 1986; Verbalis 1991). By contrast, ‘postabsorptive’ or systemic inputs provide a more sustained signal to prevent drinking when the body is in a state of fluid homeostasis, or under conditions of hypervolemia or hypo‐osmolarity, when excess water intake might exacerbate the condition (Verbalis 1991).

The speed and method for quenching thirst varies considerably among species. Some species (e.g., dog, camel, sheep, goats, deer) accurately replace fluid deficits immediately upon gaining access to water following dehydration; whereas other species (e.g. rats, humans, horse) replace fluid more gradually and may take several hours to restore body fluid volume and composition, which is termed ‘voluntary dehydration’ (McKinley 2009). This is likely due to different types of signals among species, or different emphasis on preabsorptive and postaborptive elements (Gibbs et al. 1986). In this review, we focus particularly on research from transgenic mice, which tend to replace fluid deficits rapidly (Zimmerman et al. 2016).

While fluid satiation generally refers to satiation for water, another key component for restoring fluid homeostasis is salt (NaCl), which is the major osmotic component of the extracellular fluid (ECF) (Johnson and Thunhorst 1997; McKinley and Johnson 2004). ECF volume is proportional to total body sodium; whereas plasma osmolality is regulated primarily by the ingestion and excretion of water (Geerling and Loewy 2008). An animal is not fully satiated until both water and NaCl have been replenished, particularly following hypovolemic‐induced thirst. To understand fluid satiation, we must therefore examine neuronal populations which control both water and salt intake and excretion.

For the purposes of this review, we have divided the neural circuitry underlying fluid satiation into three components: (1) Input signals; (2) Neurocircuitry within the central nervous system; and (3) Outputs.

## Input Signals

**Figure 1:**
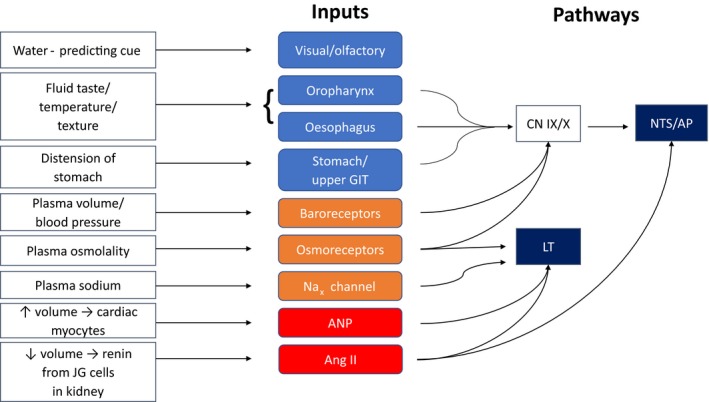
A schematic figure demonstrating peripheral inputs which modulate fluid intake. *Inputs*: Blue boxes, preabsorptive inputs; orange boxes, postabsorptive inputs; red boxes, hormonal signals. ANP, atrial natriuretic peptide; CN IX/X, cranial nerves IX (glossopharyngeal) and X (vagus); NTS, nucleus of the solitary tract; AP, area postrema; LT, lamina terminalis.

The input signals for terminating water ingestion are not well understood, but likely involve a combination of preabsorptive and postabsorptive factors (Gibbs et al. 1986) (Fig.**** 1).

### Preabsorptive inputs

Recent calcium imaging studies reveal that certain brain regions receive rapid signaling about fluid status. Drinking water rapidly inhibits neuronal calcium activity in subfornical (SFO) thirst‐related neurons, even before plasma osmolality or volume have been restored. The input signal likely arises from receptors in preabsorptive regions which send projections to the brain (Zimmerman et al. 2016, 2017). Although the precise receptors and neuronal pathways remain to be identified, studies suggest that the preabsorptive inputs can be divided into the following: (1) predictive cues; (2) oropharyngeal inputs; and (3) oesophageal and gastric inputs.

#### Predictive cues

Placing a water bottle in the cage of thirsty mice rapidly inhibits calcium activity in vasopressin‐expressing neurons in the supraoptic nucleus, even before drinking has commenced, suggesting these neurons receive rapid signaling about water‐predicting cues, perhaps via visual or olfactory inputs (Mandelblat‐Cerf et al. 2017). These inputs have not been comprehensively studied, but prepare the body for drinking and anticipate the amount of fluid required to restore homeostasis.

#### Oropharyngeal inputs

Inputs from the oral cavity and pharynx also signal fluid satiation. Gargling water can inhibit the sensation of thirst in dehydrated humans for about 15 min, suggesting oropharyngeal receptors mediate a transient sensation of fluid satiation (Seckl et al. 1986). Both water and hypertonic saline (NaCl) decrease thirst, suggesting that the type of fluid does not change the sensation of fluid satiation, at least initially; however, hypertonic saline relieves thirst only transiently, with the thirst sensation returning within an hour (Seckl et al. 1986; Zimmerman et al. 2016). However, the oropharyngeal cavity does detect a difference between fluid and food, with fluids providing a rapid inhibitory signal to thirst, but not food (Verbalis 1991).

Cold temperatures in the mouth, such as ice chips or a cold metal spout, are also experienced as more thirst‐quenching than warmer temperatures, suggesting temperature receptors in the oral cavity regulate fluid satiation (Zimmerman et al. 2016). In addition, a recent paper reported that the taste of water was detected by acid‐sensing taste receptor cells expressing the cation channel PKD2LI (polycystic kidney disease 2‐like 1 protein) in the mouth, which may contribute to the preabsorptive signal for fluid satiation (Zocchi et al. 2017); however, these cells do not appear to encode the actual signal for fluid satiation, because optogenetically activating these cells leads to continuous and unimpeded licking of an empty bottle in water‐deprived animals (Zocchi et al. 2017).

#### Oesophageal and gastrointestinal inputs

Studies conducted with open esophageal or gastric fistulae reveal that animals drink considerably more water than required for their fluid deficit (termed ‘sham‐drinking’), and many animals will continue to drink until they appear fatigued, suggesting that the effort of drinking and swallowing water per se is insufficient to satiate thirst (Towbin 1949; Verbalis 1991). Inputs arising from the stomach appear to relay signals about mechanical distension, because cumulative volume correlates highly with the sensation of fluid sensation, with substances such as air or hypertonic saline injection, or even balloon inflation, causing a similar effect on fluid inhibition (Towbin 1949; Hoffmann et al. 2006).

If an animal with an open gastric fistula is co‐infused with water into its intestine, there is a dose‐related decrease in sham‐drinking, but not a complete halt, suggesting that postabsorptive receptors mediate part, but not all, of the fluid satiation effect (Wood et al. 1980). If an animal is infused intravenously with water (i.e., directly into the systemic circulation, bypassing the gastrointestinal system), there is a decrease in sham drinking; however, not as great as would be expected if systemic factors were the only factors responsible for terminating fluid intake (Wood et al. 1980).

Overall, there appears to be a combination of oropharyngeal, esophageal and gastric signals in an appropriate temporal sequence for fluid satiation to be achieved in the short term. While the precise receptors and neuronal projections remain to be identified, it is likely that the preabsorptive signals are carried via the vagal nerve, because capsaicin treatment, which damages vagal afferent nerves from the gastrointestinal tract, leads to lack of fluid satiation initially (Curtis and Stricker 1997) and hepatic vagotomy causes dehydrated rats to overdrink (Smith and Jerome 1983).

### Postabsorptive inputs

In the longer term, absorption of fluid into the systemic circulation and reduction of plasma osmolality restores fluid homeostasis, which is detected by postabsorptive signals, such as baroreceptors (blood volume/pressure sensors), osmoreceptors (blood osmolality sensors), and sodium sensors, which signal to prevent or diminish further fluid and salt intake. For example, increased blood pressure inhibits drinking, whereas lowered blood pressure enhances drinking (Thunhorst et al. 1993). These postabsorptive inputs have been previously reviewed in detail (Wood et al. 1980; Gibbs et al. 1986; Johnson and Thunhorst 1997; McKinley and Johnson 2004; Bourque 2008), and are critical for decreasing or preventing fluid and salt intake when water is in excess, that is, hypervolaemia, or when the body's sodium/water ratio is altered, such as by hypo‐osmolarity (Verbalis 1991).

Postabsorptive signals also include the renin–angiotensin–aldosterone hormonal system which promotes water and salt intake under conditions of hypovolemia (Fitzsimons 1998) and atrial natriuretic peptide (ANP), which is released from cardiac atrial myocytes under conditions of hypervolemia and can inhibit fluid intake after pharmacological injection, suggesting a role in fluid satiation (Antunes‐Rodrigues et al. 1985).

## Neurocircuitry within the Central Nervous System

**Figure 2:**
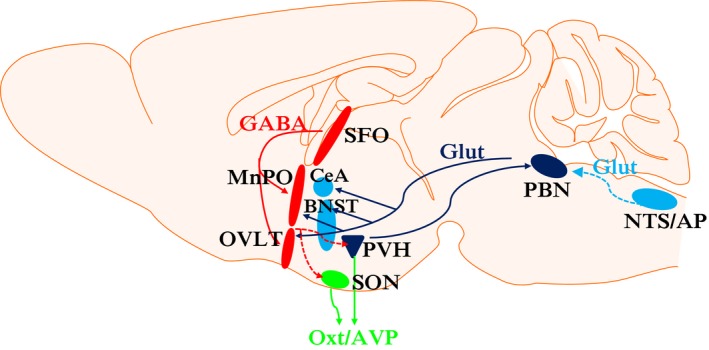
Schematic diagram showing neural circuitry which decreases fluid intake. Their output projections to centres involved in conscious perception or behavioural functions are currently unknown. Dark blue, glutamatergic neurons; dark blue connectors , glutamatergic projections; light blue, presumably glutamatergic neurons; dotted light blue connectors, presumably glutamatergic projection; red, GABAergic neurons; red connectors, GABAergic projections; dotted red connectors, presumably GABAergic connectors; green, paraventricular hypothalamic and supraoptic nuclei; green line, projections to posterior pituitary. AP, area postrema; AVP, vasopressin; BNST, bed nucleus of the stria terminalis; CeA, central nucleus of the amygdala; Glut, glutamatergic; MnPO, median preoptic nucleus; NTS, nucleus of the solitary tract; OVLT, organum vasculosum of the lamina terminalis; Oxt, oxytocin; PBN, parabrachial nucleus; PVH, paraventricular nucleus of the hypothalamus; SFO, subfornical organ; SON, supraoptic nucleus. Sagittal mouse brain outline from Motifolio toolkit.

The amount of fluid ingested is rapidly relayed from peripheral inputs to the central nervous system. While the precise brain regions underlying the conscious signal for fluid satiation remains unknown, studies have revealed multiple neuronal populations that prevent or decrease fluid intake (Fig.**** 2). Many brain regions were initially identified by lesion, pharmacological, electrophysiological, and immunohistochemical studies of Fos expression (McKinley et al. 1994; Johnson and Thunhorst 1997; McKinley and Johnson 2004). More recently, genetically encoded techniques, including optogenetics, DREADDs (designer receptors exclusively activated by designer drugs), and calcium imaging, have provided more finely tuned characterization of specific cell types, their properties and projections.

Several recent reviews have outlined our current understanding of the neural circuitry for thirst (Leib et al. 2016; Zimmerman et al. 2017), which frequently overlaps with the neural circuits of fluid satiation. In this review, we focus particularly on the hindbrain regions, which play a considerable role in mediating fluid satiation, compared to forebrain structures such as the subfornical organ (SFO), which tend to facilitate thirst and sodium appetite. However, more recent genetically encoded studies have suggested a more complex interplay of signals within and between brain regions, with certain neuronal subsets in each brain region mediating opposite effects to adjacent neurons, e.g., GABAergic neurons in the SFO inhibit fluid intake (Johnson and Thunhorst 1997; Oka et al. 2015).

### Hindbrain regions

#### Nucleus of solitary tract (NTS) and area postrema (AP)

The NTS and AP are adjacent structures in the hindbrain which receive peripheral input regarding the body's fluid status (as well as multiple other forms of viscerosensory input, such as metabolic and respiratory status) (Hyde and Miselis 1984; Price et al. 2008). These brain regions appear to play a key role in mediating fluid satiation: lesions to the medial NTS and AP region increase water and salt intake (Edwards and Ritter 1982; Hyde and Miselis 1984; Ohman and Johnson 1989); whereas activating NTS/AP neurons, using the stimulatory DREADD, hM_3_Dq, suppresses fluid intake (Ryan et al. 2017). This region does not appear to be selective for fluid intake, however, because activating NTS/AP neurons also decreases food and salt (NaCl) intake (Ryan et al. 2017). Further investigations are now required to identify different neuronal populations in the NTS/AP region, their expression patterns and their distinct functions. Notably, a selective neuronal marker, HSD2, has been identified in a subset of NTS neurons, which *increases* NaCl intake (Geerling and Loewy 2009; Jarvie and Palmiter 2017), demonstrating a complex interplay of signals within the NTS.

#### Parabrachial nucleus (PBN)

A major projection site for NTS neurons is the hindbrain parabrachial nucleus (PBN), which also plays a key role in inhibiting fluid and NaCl intake (Menani et al. 2014). For example, NaCl intake was substantially increased after injecting several antagonists into the PBN, including: methysergide, a nonselective serotonin receptor antagonist; proglumide, a nonselective cholecystokinin receptor antagonist; and *α*‐helical CRF_9‐41_, a nonselective corticotropin‐releasing factor (CRF) receptor antagonist; although only when accompanied by additional facilitatory treatments that promote fluid intake, such as angiotensin II (Menani et al. 2014). These antagonist injections also increased water intake, although this was generally less than the NaCl intake (Menani et al. 2014).

Certain other compounds, such as muscimol, a GABA_A_ receptor agonist or *β*‐endorphin, a *μ*‐opioid agonist, increase both NaCl and water intake when injected into the PBN, without the need for additional facilitatory treatments. This suggests that there are separate, although likely overlapping, neuronal populations within the lateral PBN, some which primarily limit NaCl intake when accompanied by facilitatory treatments, whereas others limit both NaCl and water intake without the need for facilitatory treatments (Menani et al. 2014).

#### Oxytocin receptor‐expressing neurons in the parabrachial nucleus (Oxtr^PBN^neurons)

Recently, Ryan et al. (2017) discovered a subset of oxytocin‐receptor expressing neurons in the PBN (Oxtr^PBN^ neurons), which selectively decreased noncaloric fluid intake, but not food intake after fasting, or salt intake after salt depletion. Calcium imaging studies demonstrated that Oxtr^PBN^ neurons rapidly increased calcium activity when drinking water after 24‐h dehydration, but not after drinking from an empty bottle or ingesting a high‐caloric liquid diet, Ensure^®^, indicating a selective role for Oxtr^PBN^ neurons in fluid satiation (Ryan et al. 2017). The rapidity of these neuronal responses suggests Oxtr^PBN^ neurons receive rapid input from preabsorptive inputs, likely via the NTS (Ryan et al. 2017).

By contrast, inactivation of Oxtr^PBN^ neurons increased NaCl intake, but only after mice were subjected to dehydration or hypertonic saline injection (Ryan et al. 2017). These results suggest that Oxtr^PBN^ neurons decrease fluid intake to varying degrees depending upon the level of activation, with baseline activation decreasing NaCl intake (as indicated by the inactivation studies), while more robust activation suppresses both water and NaCl intake.

The effect of inactivating Oxtr^PBN^ neurons is similar to intra‐PBN injections of methysergide (nonselective serotonin receptor antagonist) and proglumide (nonselective CCK receptor antagonist) (Menani et al. 2014), suggesting that Oxtr^PBN^ neurons may co‐express receptors for these other compounds; however, further immunohistochemical studies are required to validate co‐expression. Furthermore, it has been suggested that various types of inhibitory inputs may converge on the PBN which each use different neurochemical codes. For example, input from arterial baroceptors might use serotonin as an inhibitory signal, whereas concentrated salt solutions might use CCK as a signal; however, further experimentation will be required to tease these inputs apart (Menani et al. 2014).

Recent studies have demonstrated that activating another Oxtr‐expressing population in the hypothalamic arcuate nucleus (Oxtr^ARC^) decreases food intake (Fenselau et al. 2017), suggesting that different Oxtr‐expressing neural populations control caloric versus noncaloric substances. Investigating different Oxtr‐expressing neuronal populations may provide critical insight into differentiating these circuits.

### Forebrain regions

#### Amygdala and Bed Nucleus of Stria Terminalis (BNST)

There are major ascending neural projections from hindbrain regions (NTS and PBN) to both the central nucleus of the amygdala (CeA) and BNST, which are heavily interconnected and which receive inputs from multiple other visceral and somatic sensory regions, suggesting an integrative centre (Sawchenko 1983; Johnson et al. 1999; Dong et al. 2001; Ryan et al. 2017). These regions are implicated as key regulators of NaCl intake: electrolytic lesions of the CeA and BNST in rats markedly decrease salt intake, but not water, food or 5% sucrose (Galaverna et al. 1992; Zardetto‐Smith et al. 1994; Johnson et al. 1999); and sodium depletion robustly increases Fos expression in the CeA and BNST (Johnson et al. 1999).

It is likely that NaCl intake by the CeA is mediated by endogenous opioids, given that injection of μ‐opioid receptor antagonists into the CeA significantly decreases NaCl intake appetite in mice (Smith et al. 2016). Studies also suggest that the PBN and CeA interact to control NaCl intake: deactivating the PBN increases NaCl intake, which is blocked by intra‐CeA injection of opioid receptor antagonists (Andrade‐Franze et al. 2017). More detailed studies on selective neuronal populations within the CeA and BNST are now required to understand their specific roles in fluid and salt intake.

#### Paraventricular hypothalamic nucleus (PVH) and supraoptic nucleus (SON)

The PVH and SON play key roles in regulating osmolarity (Bourque 2008; Stachniak et al. 2014). Both nuclei contain magnocellular (large) neurons which express vasopressin and oxytocin, and project to the posterior pituitary to secrete these fluid‐regulating peptides peripherally. The PVH also contains parvocellular neurons which project within the central nervous system (Swanson and Kuypers 1980). In terms of peripheral functions, vasopressin promotes water retention via the kidney; while oxytocin causes natriuresis in rats (Verbalis et al. 1991). Under conditions of increased osmolality, oxytocin and vasopressin act in concert to restore fluid homeostasis by increasing both antidiuresis and sodium excretion; however, under conditions of hypovolemia (where it is important to suppress sodium excretion while enhancing antidiuresis), angiotensin II is released, which potentiates vasopressin neuron firing but suppresses oxytocin neuron firing, enabling overall retention of fluid (Stachniak et al. 2014).

Calcium imaging of vasopressin neurons in the SON that project to the posterior pituitary (VP_pp_ neurons) revealed that during elevated osmolality, VP_pp_ neurons decrease activity within seconds after being presented with water‐predicting cues, with activity decreasing even further upon drinking water, suggesting a method of anticipating correction of osmolality (Mandelblat‐Cerf et al. 2017). By contrast, food availability following food restriction rapidly increased VP_pp_ neuron activity within seconds of feeding onset, implying these neurons are responsive to anticipated increases in osmolality (Mandelblat‐Cerf et al. 2017).

Oxytocin‐expressing neurons in the PVH (Oxt^PVH^) decrease water intake when activated, although the effect is relatively mild compared to Oxtr^PBN^ activation. This milder effect is likely due to activating only a subset of Oxtr^PBN^ neurons (Ryan et al. 2017); alternatively, it may be due to an interaction between the central and peripheral effects of oxytocin, which have opposite effects on blood pressure; that is, peripheral oxytocin decreases blood pressure (Gutkowska and Jankowski 2008), which may increase fluid intake; whereas central oxytocin increases blood pressure via interactions with nesfatin and corticotrophin‐releasing factor (CRF) (Yosten and Samson 2014), which may decrease fluid intake.

The effect of vasopressin‐expressing neurons in the PVH has not been tested directly, although a recent study demonstrated that activating vasopressin neurons in the suprachiasmatic nucleus increased water intake, particularly anticipatory thirst prior to sleep (Gizowski et al. 2016).

#### Lamina terminalis

A major forebrain region which coordinates fluid intake is the lamina terminalis, which comprises three nuclei: the subfornical organ (SFO), the organum vasculosum of the lamina terminalis (OVLT) and the median preoptic nucleus (MnPO) (Johnson and Thunhorst 1997; McKinley and Johnson 2004; Zimmerman et al. 2017).

In contrast to hindbrain regions, these forebrain regions primarily signal thirst, and activation of these regions increase fluid and salt intake, which has been recently reviewed in detail (Zimmerman et al. 2017). However, subpopulations of GABAergic nerves within these regions *decrease* fluid intake, but not salt intake in salt‐depleted mice, or sucrose intake in hungry mice, suggesting they play a role in controlling fluid satiation (Oka et al. 2015).

#### Conscious appreciation of thirst

The brain regions involved in a conscious appreciation of fluid satiation remain unknown; however, fMRI studies in humans demonstrate changes in blood oxygenation level‐dependent (BOLD) response in certain brain regions during water ingestion as the subjects shifted to the nonthirsty state (Denton et al. 1999). For example, during thirst, there was a substantial increase in activity in the anterior cingulate gyrus, which had disappeared by 3 min after drinking to satiation; by contrast, there was increased activation in the mid‐cingulate area in BA 24 (Brodmann's area 24), the right precentral gyrus at BA 6, the right lateral posterior thalamus and the right superior temporal gyrus; and 14 min after drinking to satiation, a new robust activation was observed in the left cingulate gyrus, suggesting these regions play a role in mediating fluid satiation (Denton et al. 1999). In addition, neuronal tracing studies suggest that the emotional aspects of thirst may be relayed via thalamocortical pathways to the insular and cingulate cortex (Hollis et al. 2008).

In addition, advancing age appears to impact fluid intake, with elderly people drinking less water. While older and younger subjects had similar increases in blood osmolality and experienced similar levels of thirst after hypertonic infusions, older patients drank less, suggesting they arrived at fluid satiation earlier (Farrell et al. 2008). A comparison of younger and older patients revealed there was a greater reduction in anterior midcingulate cortex (aMCC) cerebral blood flow relative to water drunk in the older group, suggesting this may be an affected brain region, but whether these alterations are due to changes in primary afferent inflow or higher cortical functioning is unclear (Farrell et al. 2008).

## Outputs

The major effects of the CNS‐mediated fluid satiation response can be classified as behavioral, endocrine and autonomic (Johnson et al. 1999). The major behavioral response is to decrease or terminate fluid intake. Although the precise output brain regions for fluid satiation remain to be identified, it is likely that central pattern generator (CPG) brain regions for licking and swallowing are involved, which are likely located within or near the intermediate reticular formation (IRt) (Moore et al. 2014). This region has key connections with cranial nerves, such as IX (glossopharyngeal) and X (vagus) which supply the muscles involved in swallowing, and XII (hypoglossal) which are required for tongue movements involved in licking (Moore et al. 2014)

Other key brain regions involved in coordinating the behavioral response to fluid intake include: the substantia nigra pars reticulata (SNR), which demonstrates firing rates time‐locked to individual licks (Rossi et al., 2016); the superior colliculus which receives GABAergic projections from the SNR (Rossi et al., 2016); and the motor cortex and cerebellum (Travers et al., 1997), suggesting that these regions are part of the network coordinating fluid intake.

The endocrine and autonomic responses predominantly target the kidney to influence sodium and water excretion, such as by decreasing vasopressin secretion to limit the antidiuretic response, or by decreasing sympathetic nervous system effects on the renal tubules to prevent increased reabsorption of sodium and water (Verbalis 1991). The sympathetic responses also target the cardiovascular system; however, the immediate response to drinking water is a rapidly increased sympathetic response, which can result in increased blood pressure particularly in elderly patients or patients with autonomic failure. This response gradually diminishes over more than 1 h, by which time fluid homeostasis has been corrected (Jordan et al. 1999).

## Summary

The fluid satiation neural circuitry comprises several highly interconnected forebrain and hindbrain regions, which together act to decrease or terminate fluid intake when fluid satiated. This neural circuitry substantially overlaps with the neural circuitry for thirst, and interacts with other neural circuits, including feeding circuits and motivational circuits (Zimmerman et al. 2017). Key brain regions which mediate the fluid satiation response include the NTS, AP and PBN in the brainstem; the lamina terminalis; the CeA and BNST; and the hypothalamic PVH and SON (Fig.**** 2). Inputs to the fluid satiation neural circuit arise from oropharyngeal/upper gastrointestinal receptors, osmoreceptors, baroreceptors and peripheral hormones, which convey signals about changes in fluid volume and osmolality (see Fig.**** 1). Brain regions involved in the behavioral outputs of this circuit remain to be identified, but likely involve brain regions which mediate conscious, motivational and motor responses to decrease or terminate fluid intake.

## Future Directions

While key brain regions have already been identified, an overarching goal in understanding fluid satiation is to complete the neural circuit, tracing from input through to output, in order to understand the different components modulating fluid satiation. An essential part of understanding the fluid satiation neural circuitry will be to chemically phenotype each distinct neuronal population, which can then allow selective manipulation using genetically‐encoded technologies.

To understand how fluid intake is coordinated, it will also be important to understand how the fluid‐related neural circuitry interacts with the neural circuitry for other ingestive behaviors, for example, how feeding circuits interact with fluid intake circuits to increase thirst after feeding (Zimmerman et al. 2016). In addition, thirst is a highly motivated response, so it will be important to understand how the fluid circuits impact motivational and reward circuits. Several studies have suggested that salt consumption may decrease anxiety‐related behaviors and stimulate reward‐related neural circuits, which can be investigated in further detail (Liedtke et al. 2011; Krause et al. 2017).

Overall, genetically encoded techniques for analysing neural circuits are enabling novel insights into the fluid satiation neural circuitry, which is essential for understanding fluid homeostasis. Clinically, this is important for better managing blood pressure and hemodynamic stability, and fluid overload in conditions such as cardiac failure, renal failure, and cirrhosis with ascites, as well as overdrinking in conditions such as psychogenic polydipsia. By using genetically encoded techniques, we expect further novel insights into the fluid satiation neural circuitry in the coming years.

## Conflict of Interest

I have no conflicts of interest to disclose.
